# Effect of weight loss intervention on assisted reproductive outcomes in women with obesity and infertility: a retrospective cohort study

**DOI:** 10.3389/fendo.2026.1829965

**Published:** 2026-05-20

**Authors:** Hua Dai, Qifeng He, Yongxin Liu, Ling Wu, Hongmei Wang

**Affiliations:** 1 Reproductive Medicine Center of Yancheng Maternal and Child Health Hospital Affiliated to Yangzhou University Medical College, Yancheng, Jiangsu, China; 2 Obstetrics and Gynecology Department of Huai’an Clinical Medical College of Jiangsu University, Huaian Hospital of Huaian City, Huaian, Jiangsu, China; 3 Obstetrics and Gynecology Department of Yancheng Maternal and Child Health Hospital Affiliated to Yangzhou University Medical College, Yancheng, Jiangsu, China; 4 Nursing Department of Yancheng Maternal and Child Health Hospital Affiliated to Yangzhou University Medical College, Yancheng, Jiangsu, China

**Keywords:** assisted reproductive technology, live birth rate, retrospective cohort study, weight loss intervention, women with obesity and infertility

## Abstract

**Objective:**

This retrospective cohort study aimed to evaluate whether a goal-directed weight loss intervention prior to assisted reproductive technology (ART) improves live birth rates compared with immediate *in vitro* fertilization (IVF) without weight loss in women with obesity and infertility (body mass index [BMI] ≥30 kg/m²).

**Methods:**

A total of 308 women with obesity and infertility who underwent ART at Yancheng Maternal and Child Health Hospital between January 2022 and June 2024 were included. After excluding those with missing covariates and performing 1:1 propensity score matching (PSM), 58 patients were assigned to the Target-oriented Group (comprehensive weight loss intervention, including low-calorie diet, physical activity enhancement, and orlistat (120 mg three times daily) as a pharmacological adjunct to reduce fat absorption, aiming to achieve an 8% weight reduction) and 58 to the Standard Lifestyle Group (general lifestyle advice without specific weight loss targets). Because the weight-loss program inherently delayed ART initiation by approximately 16 weeks, a 16-week landmark analysis was performed to align the exposure assessment window between groups and minimize immortal time bias; only women who remained under active follow-up and were event-free at week 16 were included in the analysis.

**Results:**

After 16 weeks, the Target-oriented Group achieved a mean weight reduction of 9.96% (standard deviation [SD] 9.40), whereas the Standard Lifestyle Group showed minimal change (0.68%, SD 2.07; P<0.001). The live birth rate was significantly higher in the Target-oriented Group (39.66%, 23/58) than in the Standard Lifestyle Group (15.52%, 9/58) (risk difference: 24.1%, 95% CI: 6.8–41.5%, *P* = 0.004). In the 16-week landmark cohort, the Target-oriented Group demonstrated a higher likelihood of achieving clinical pregnancy than the Standard Lifestyle Group (adjusted hazard ratio [HR] 2.86, 95% confidence interval [CI]: 1.31–6.22). No significant between-group differences were observed in multiple pregnancy rate (3/23 [13.04%] vs. 1/9 [11.11%]), mean neonatal birth weight (3.33 ± 0.36 kg vs. 3.40 ± 0.60 kg), or neonatal complications (2/31 [6.45%] vs. 0/10 [0%]) (all P > 0.05).

**Conclusion:**

Although this retrospective study design limits causal inference, a 16-week intensive goal-directed weight loss intervention appeared to be associated with both significant weight reduction and higher live birth rates among women with obesity and infertility, in comparison with immediate IVF without prior weight management.

## 1 Introduction

With rising living standards and changes in dietary structure, the global prevalence of overweight and obesity has continued to increase, representing a major worldwide medical and social challenge. According to Chinese Body mass index (BMI) classification criteria, a BMI between 24 and <28 kg/m² is considered overweight, and a BMI ≥28 kg/m² indicates obesity. A nationwide, real-world cross-sectional study conducted in 2023, encompassing 15,770,094 Chinese adults, revealed that 34.8% were overweight and 14.1% had obesity according to Chinese BMI criteria. After age-standardization, the prevalence of obesity among women was 9.6% (1). Among women of reproductive age specifically, based on national surveillance data, the rates of overweight and obesity were 25.4% and 9.2%, respectively (2).

Accumulating evidence indicates that obesity in women is associated with dysfunction of the hypothalamic–pituitary–ovarian axis, hyperinsulinemia, hyperandrogenemia, and chronic inflammation, which can lead to menstrual irregularities, ovulatory disorders, and infertility (3, 4). Previous clinical studies have shown that the probability of natural conception may decrease with increasing BMI, though the precise nature of this dose-response relationship requires further clarification (5, 6).

Evidence from at least one systematic review suggests that effective weight loss interventions may mitigate the adverse effects of overweight and obesity on female fertility through multiple mechanisms (7). A retrospective study demonstrated that women with overweight or obesity and infertility who lost ≥10% of their body weight had higher rates of pregnancy (88% vs. 54%, P = 0.049) and live birth (71% vs. 37%, P = 0.024) compared with controls (8). Another small randomized trial involving 49 women with obesity also reported that the weight loss intervention group achieved significantly higher rates of pregnancy (48.1% vs. 13.6%, P = 0.007) and live birth (44.4% vs. 13.6%, P = 0.02) (9). In a retrospective study, 18.75% of patients with obesity who attained their preconceptional weight-loss target conceived spontaneously. For those who subsequently underwent *in vitro* fertilization (IVF), the clinical pregnancy rate was 62.5% (10).

However, whether weight loss interventions can improve assisted reproductive technology (ART) outcomes in women with obesity and infertility remains controversial (11). Some researchers propose that weight loss may enhance response to ovulation induction agents, improve ovarian stimulation outcomes, ameliorate endocrine profiles and insulin resistance, increase the number of retrieved oocytes, and improve embryo quality (12, 13). Conversely, other studies have reported no improvement in ART outcomes despite successful weight loss (11). For instance, a Nordic multicenter study including 317 women with obesity found no significant difference in overall live birth rates between the weight loss intervention group and the control group following the first IVF cycle (14). Similarly, a Dutch randomized controlled trial (RCT) published in 2016 reported no beneficial effect of weight loss intervention on the 2-year cumulative live birth rate in patients with infertility and obesity (15). Several potential explanations have been proposed for the lack of benefit observed in previous RCTs. The Dutch RCT achieved only a modest between-group weight difference of 3.3 kg, with a high discontinuation rate of 22% in the intervention group. Similarly, the Nordic RCT enrolled women with a relatively lower median BMI, potentially limiting the metabolic benefits achievable through weight loss. Insufficient magnitude of weight reduction, high heterogeneity in intervention adherence, and failure to achieve a clinically meaningful weight loss threshold (commonly cited as ≥5–10%) may collectively explain the null findings of these trials.

Given these conflicting findings, this retrospective cohort study aimed to evaluate whether a goal-directed weight loss intervention prior to ART improves live birth rates compared with immediate IVF without weight loss in women with obesity and infertility (BMI ≥30 kg/m²). We hypothesized that a structured, goal-directed weight loss intervention achieving ≥8% weight reduction would significantly improve live birth rates compared with immediate IVF initiation without weight management in women with obesity and infertility with BMI ≥30 kg/m².

## 2 Methods

### 2.1 Study design and study population

This retrospective cohort study was conducted to analyze clinical data derived from women with obesity and infertility with a BMI of ≥30 kg/m², who underwent ART procedures at Yancheng Maternal and Child Health Hospital between January 2022 and June 2024. The study was approved by the Institutional Review Board of Yancheng Maternal and Child Health Hospital (2025041). The first and corresponding authors assume responsibility for the accuracy and completeness of the data.

Eligible participants were identified according to predefined inclusion and exclusion criteria. Inclusion Criteria: (1) Age 22–40 years; (2) Indication for IVF and planned initiation of first IVF cycle; (3) BMI ≥30 kg/m². Exclusion Criteria: (1) Insulin-dependent diabetes; (2) Planned use of donor oocytes or preimplantation genetic diagnosis; (3) Partner with azoospermia; Diagnosis of binge eating disorder according to the Questionnaire on Eating and Weight Patterns–Revised (16); (4) Previous participation in a similar weight loss intervention study; (4) Missing values of relevant covariates.

### 2.2 Exposure definition and group allocation

The exposure of interest was participation in a structured pre-ART weight-loss intervention. Group assignment was based on patient preference and clinical recommendation: patients who opted for immediate IVF without structured weight loss were assigned to the Standard Lifestyle Group, while those willing to undergo intensive weight management prior to ART were allocated to the Target-oriented Group.

To address immortal time bias arising from the protocol-mandated delay in ART initiation in the Target-oriented Group, we implemented a 16-week landmark analysis. The time origin (time zero) was defined as the date of first clinic attendance when the patient confirmed her group allocation decision. For the Target-oriented Group, the 16-week weight loss intervention was completed prior to ART initiation; IVF was therefore commenced approximately 16–20 weeks after time zero.

Only patients who remained under active follow-up and had not experienced the outcome event (clinical pregnancy) by week 16 were included in the landmark cohort, with the time origin reset to the 16-week landmark date for both groups, thereby aligning the exposure assessment windows. The primary outcome of live birth rate was assessed as a cumulative binary outcome over all completed IVF cycles during the follow-up period. Censoring for the time-to-event analyses occurred at treatment discontinuation, loss to follow-up, or end of the study period, whichever came first. To minimize selection bias, propensity score matching was performed to balance baseline characteristics.

### 2.3 Clinical management and intervention protocols

The clinical management process consisted of two sequential phases: a lifestyle intervention phase followed by standardized infertility treatment. Based on the study objectives, patients were categorized into the following two groups.

#### 2.3.1 Target-oriented group

This group received comprehensive interventions, including increased physical activity and calorie restriction, aiming to achieve an 8% weight reduction. The target of ≥8% weight reduction was selected based on evidence that a minimum 5% loss is sufficient to restore insulin sensitivity and ovarian function (17), while reductions ≥10% confer additional reproductive benefit (8), positioning 8% as a clinically pragmatic and evidence-based intermediate threshold consistent with the 2021 ASRM Practice Committee recommendations for preconception weight management in women with obesity (18).

##### 2.3.1.1 Dietary intervention

The 16-week intervention comprised two phases (1): a 12-week low-calorie liquid diet (LCD) phase providing 880 kcal per day, and (2) a 4-week transition phase with individualized dietitian support for gradual reintroduction of solid foods while maintaining caloric restriction. Throughout the intervention, participants received nutritional counseling and used Nutrisystem^®^ meal replacement products (three meals per day) to control food intake and calorie consumption. They also took orlistat to reduce fat absorption.

The diet plan aimed to provide approximately 1100 kcal/day, with a macronutrient composition of 30% protein, 45% carbohydrates, and 25% fat. Depending on individual needs, participants could consume an additional 100 kcal/day, resulting in a total intake of approximately 1200 kcal/day, consistent with the Look AHEAD trial (19). Daily intake included two servings of fruits, three servings of vegetables, and two servings of low-fat dairy products to ensure nutritional adequacy.

This dietary protocol was part of a formally standardized clinical program implemented at our center; intervention details and patient adherence were documented in structured medical records and extracted for the purposes of this retrospective analysis.

##### 2.3.1.2 Physical activity intervention

Participants were required to increase their average daily step count by 500 steps per week until reaching the target of 10,000 steps per day, which was maintained throughout the study period.

#### 2.3.2 Standard lifestyle group

The Standard Lifestyle Group was instructed to maintain their usual daily routine and general healthy living habits without any specific weight loss intervention. No individualized caloric targets, structured dietary plans, prescribed physical activity regimens, or weight-loss medications were provided to participants in this group. They were merely advised to follow routine, general health recommendations for weight maintenance in daily life, without additional behavioral or lifestyle modifications aimed at weight reduction.

Because group assignment was based on patient preference, potential confounding by indication was considered. Women who chose to undergo the weight loss intervention may differ systematically from those who chose immediate IVF in unmeasured characteristics such as health-related motivation, adherence behavior, and socioeconomic factors. Propensity score matching was employed to mitigate measured confounding, but residual confounding from unmeasured factors cannot be excluded.

### 2.4 IVF treatment

All patients underwent pituitary downregulation with a gonadotropin-releasing hormone agonist (GnRH-a), followed by individualized ovarian stimulation with recombinant follicle-stimulating hormone (FSH-α; Gonal-f^®^, Merck KGaA) at a dose range of 112.5–300 international units (IU)/day. Follicular development was monitored by serum estradiol levels and/or transvaginal ultrasound. When follicles reached an appropriate size, ovulation was triggered with recombinant human chorionic gonadotropin (α-hCG; Ovidrel^®^, Merck KGaA).

Approximately 36 hours later, oocyte retrieval was performed via transvaginal ultrasound-guided puncture. Fertilization was carried out using conventional IVF techniques. For male factor infertility, intracytoplasmic sperm injection (ICSI) was employed. Embryo transfer (ET) was typically performed at the cleavage stage, namely Day 2 or Day 3.

Cleavage-stage embryos were graded according to the Istanbul consensus grading system. Grade I embryos, defined as ≥6 cells with ≤10% fragmentation, and Grade II embryos were preferentially selected for transfer.

The number of embryos transferred was determined by the treating physician based on the patient’s age, embryo quality, and uterine conditions, in accordance with institutional guidelines. Single embryo transfer (SET) was performed in 29 patients (50.0%) in the Target-oriented Group and 31 patients (53.4%) in the Standard Lifestyle Group, while double embryo transfer was performed in the remaining patients. This difference was not statistically significant (P = 0.71).

Luteal phase support was provided with vaginal progesterone starting from the day of oocyte retrieval. Vaginal progesterone, 400 mg twice daily, was continued for 12 weeks in cases of confirmed clinical pregnancy, or until 14 days post-transfer if pregnancy was not achieved. Clinical pregnancy was defined as a serum hCG level >5 IU/L and was confirmed by transvaginal ultrasound at approximately 7 weeks of gestation.

### 2.5 Outcome measures

The primary outcome was live birth. Live birth was defined as the delivery of at least one live-born infant at ≥22 weeks of gestation, consistent with the ICMART/WHO international glossary of infertility and fertility care terminology.

Secondary outcomes included the following: (1) Clinical pregnancy rate: proportion of patients with ultrasonographically confirmed intrauterine gestational sac with fetal cardiac activity per patient who underwent embryo transfer; (2) Multiple pregnancy rate: proportion of clinical pregnancies with ≥2 fetal cardiac poles; (3) Miscarriage rate: pregnancy loss confirmed by ultrasound or histology before 22 weeks of gestation, per clinical pregnancy; (4) Neonatal birth weight: weight recorded within 24 hours of delivery, in kilograms; (5) Neonatal complications: a composite of preterm birth (<37 weeks), small for gestational age (<10th percentile for gestational age and sex), admission to neonatal intensive care unit (NICU), congenital anomalies, and perinatal death; (6) Magnitude of weight change: percentage change in body weight from baseline to the last measurement before or at oocyte retrieval.

### 2.6 Sample size calculation

Propensity score matching (1:1 ratio) was used to match patients between the Target-oriented Group and the Standard Lifestyle Group. Finally, 58 patients were included in the Target-oriented Group and 58 patients in the Standard Lifestyle Group, based on which the power analysis was performed. According to the incidence rates of 15.52% and 39.66% in the two groups, the GofChisquarePower method was applied for power analysis. The results showed that the statistical power was 83.83% at a significance level of α = 0.05.

### 2.7 Statistical analysis

Propensity score matching was performed using logistic regression including age, duration of infertility, baseline BMI, educational level, causes of infertility, previous treatment history, and previous live birth history as covariates. Patients were matched 1:1 using nearest neighbor matching with a caliper width of 0.1 times the standard deviation of the logit of the propensity score. Balance was assessed using standardized mean differences (SMD < 0.2 indicating adequate balance).

Continuous variables were presented as mean ± standard deviation or median (interquartile range). Inter-group comparisons were conducted using the Student’s t-test (for normally distributed data) or Mann-Whitney U test (for non-normally distributed data). Categorical variables were expressed as counts (percentages), and comparisons were made using the chi-square test or Fisher’s exact test, as appropriate. Time-to-pregnancy was analyzed using Cox proportional hazards regression. The event was the first occurrence of clinical pregnancy, defined as the ultrasonographic detection of at least one intrauterine gestational sac with evidence of fetal cardiac activity, confirmed at approximately 7 weeks of gestation, consistent with the ICMART/WHO definition. To align the exposure assessment window between groups, analyses were performed in the 16-week landmark cohort, including only women who remained under active follow-up and were not pregnant by week 16. The time origin was the landmark date (16 weeks after cohort entry), and participants were followed until clinical pregnancy or censoring. Censoring occurred at treatment discontinuation, loss to follow-up, or the end of the study period, whichever came first.

Hazard ratios (HRs) and 95% confidence intervals (CIs) were estimated, with the Standard Lifestyle Group as the reference. Secondary safety outcomes were compared using the chi-square test or Fisher’s exact test. For primary and key secondary outcomes, risk differences (RD), risk ratios (RR), and their corresponding 95% confidence intervals (95% CI) were calculated. All data were analyzed using R software (Version 4.3.2). A two-tailed P-value < 0.05 was considered statistically significant.

## 3 Results

### 3.1 Participant characteristics

**Figure 1:**
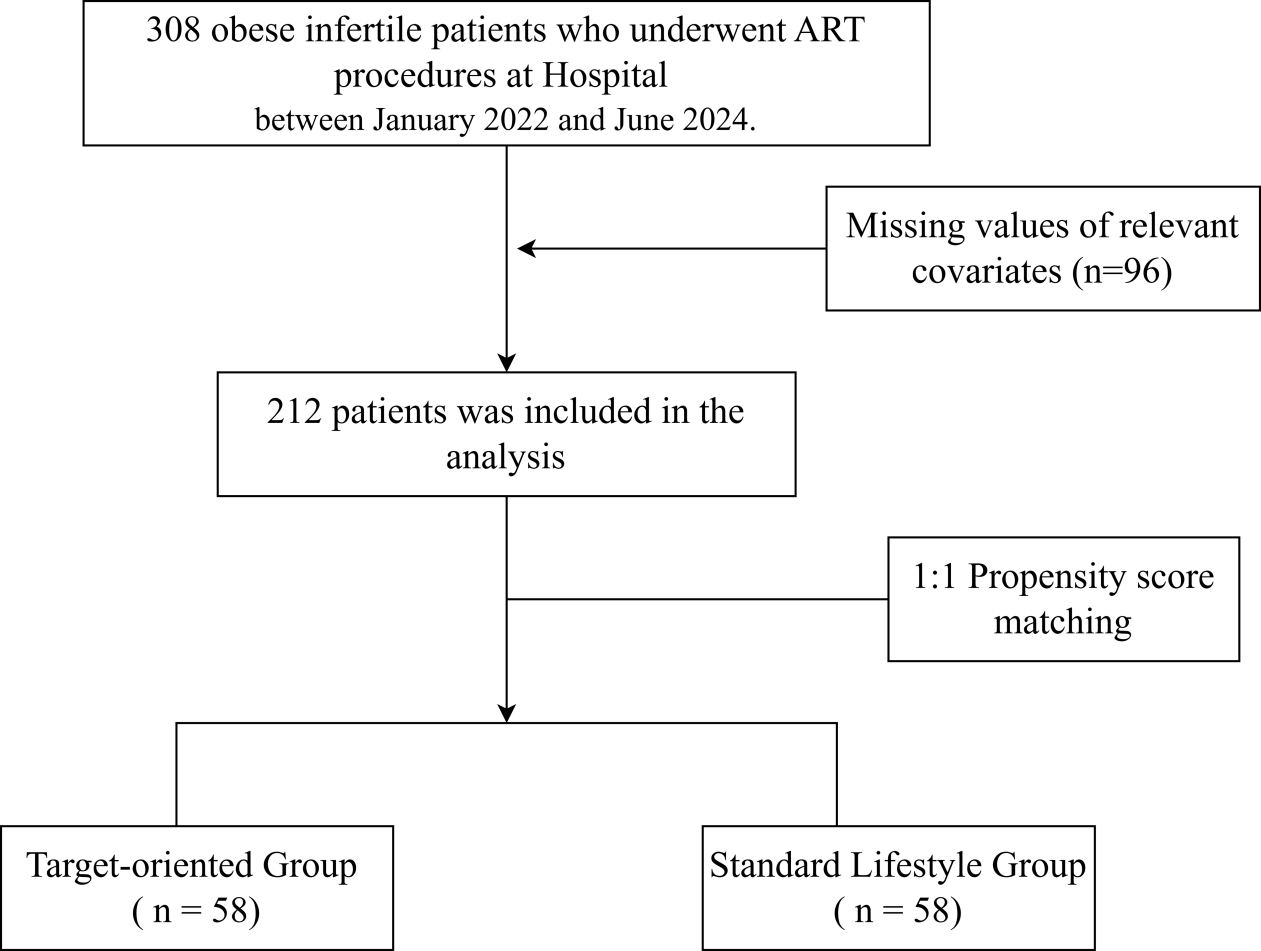
Flow diagram.

A total of 308 women with obesity and infertility who underwent ART procedures at Yancheng Maternal and Child Health Hospital between January 2022 and June 2024 were screened for eligibility. Of these, 116 patients received weight loss advice and were initially allocated to the Target-oriented Group, while the remaining 192 patients who did not pursue structured weight management were allocated to the Standard Lifestyle Group. In the Target-oriented Group, 31 patients were subsequently excluded (protocol withdrawal: n = 16; loss to follow-up: n = 10; other reasons: n = 5), yielding 85 eligible patients. In the Standard Lifestyle Group, 65 patients were excluded (pregnancy occurring before 16 weeks: n = 32; initiation of targeted weight loss intervention during follow-up: n = 22; loss to follow-up: n = 7; other reasons: n = 4), yielding 127 eligible patients. The resulting 212 patients (85 in the Target-oriented Group and 127 in the Standard Lifestyle Group) were subjected to 1:1 propensity score matching (PSM), after which 58 patients were retained in each group for the primary analysis. The complete trial flow is presented in **Figure 1**.

**Figure 2:**
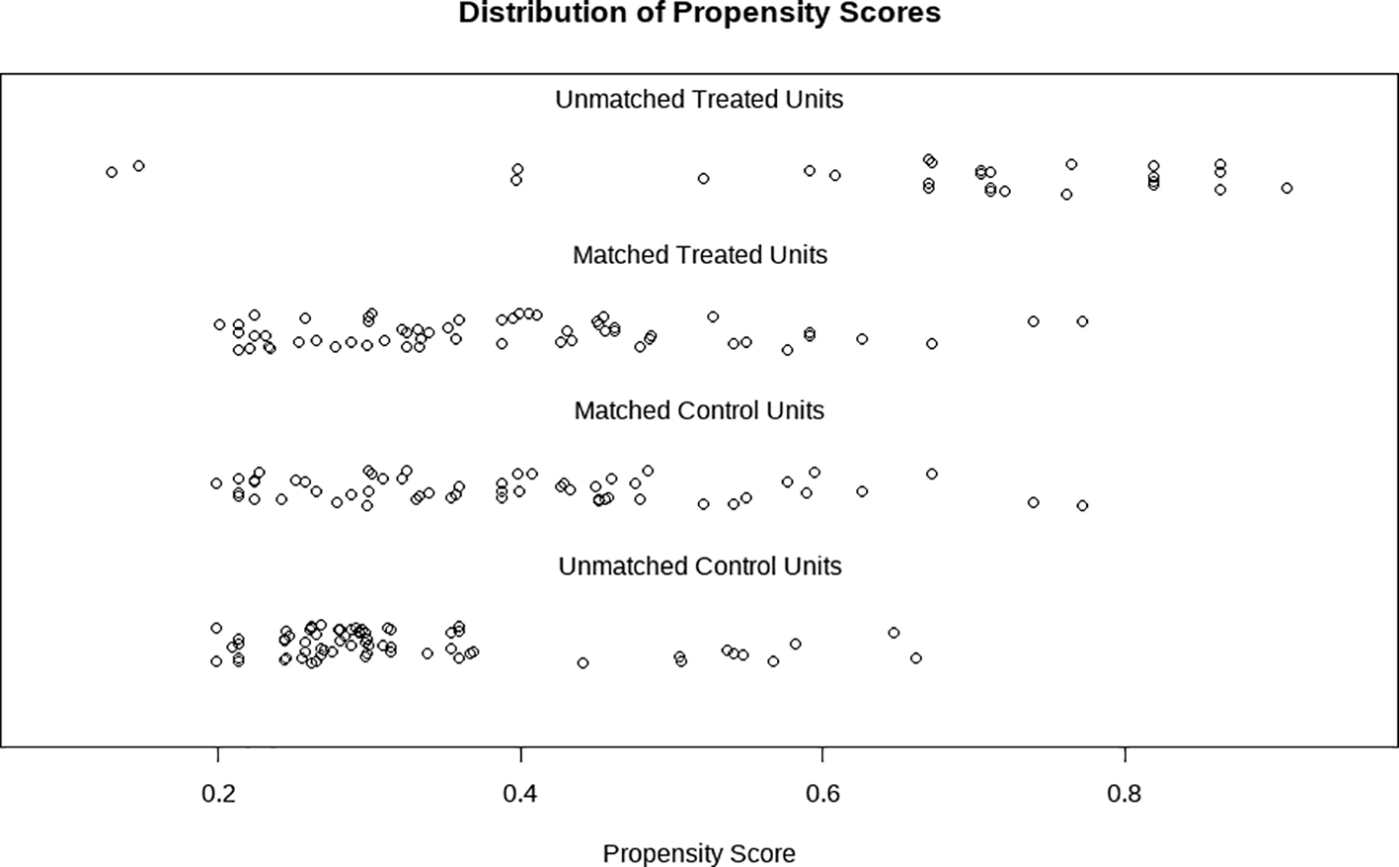
Distribution of propensity scores before and after propensity score matching.

Baseline characteristics included age, duration of infertility, baseline BMI, type of infertility, and prior pregnancy history. A total of 212 eligible patients were included in this study. Among them, the BMI of the Target-oriented Group was lower than that of the Standard Lifestyle Group, and the difference between the two groups was statistically significant (p < 0.001). After propensity score matching, 116 patients were retained. There were no statistically significant differences in baseline characteristics between the two groups (all p > 0.05), and the standardized mean differences (SMDs) were all less than 0.2, indicating that the differences between the two groups were negligible. Detailed results are shown in **Table 1**. **Figure 2** has been added to the revised manuscript showing density plots of the propensity score distributions in both groups before and after matching. The figure demonstrates substantial overlap after matching, confirming adequate balance.

**Table 1:** Baseline characteristics of unmatched and 1:1 propensity score–matched patients between the target-oriented group and the standard lifestyle group.

Characteristics	Before PS-matching				After PS-matching			
	Target-oriented group(n=85)	Standard lifestyle group (n=127)	*p*	SMD	Standard lifestyle group (n=58)	Target-oriented group (n=58)	*p*	SMD
Age(years)	32.41 ± 3.90	31.74 ± 3.59	0.199	0.172	31.62 ± 3.39	31.86 ± 3.57	0.709	0.062
Duration of Infertility(months)	31.8 (25.90, 39.90)	31.5 (25.75, 37.20)	0.603	0.075	31.4 (24.42, 40.43)	31.5 (25.00, 39.72)	0.486	0.105
BMI(kg/m^2^)	37.3 (32.22, 39.86)	39.77 (37.12, 40.58)	<0.001	0.533	38.54 (35.63, 40.50)	38.66 (36.68, 40.16)	0.919	0.024
Educational Level			0.228	–			0.629	–
College or University	55 (64.71)	86 (67.72)		-0.063	43 (74.14)	40 (68.97)		-0.108
High School or Below	12 (14.12)	9 (7.09)		0.202	6 (10.34)	5 (8.62)		-0.050
Postgraduate Degree	18 (21.18)	32 (25.20)		-0.098	9 (15.52)	13 (22.41)		0.169
Causes of Infertility			0.545	–			0.597	–
Polycystic Ovary Syndrome	18 (21.18)	32 (25.20)		-0.098	17 (29.31)	14 (24.14)		-0.127
Tubal Factor	37 (43.53)	44 (34.65)		0.179	17 (29.31)	22 (37.93)		0.174
Endometriosis	16 (18.82)	31 (24.41)		-0.143	12 (20.69)	14 (24.14)		0.088
Other	14 (16.47)	20 (15.75)		0.019	12 (20.69)	8 (13.79)		-0.186
Previous Treatment History			0.632	–			0.737	–
None	32 (37.65)	41 (32.28)		0.111	19 (32.76)	23 (39.66)		0.142
One Treatment	35 (41.18)	53 (41.73)		-0.011	25 (43.1)	22 (37.93)		-0.105
More than One Treatment	18 (21.18)	33 (25.98)		-0.118	14 (24.14)	13 (22.41)		-0.042
Previous Live Birth History			0.693	–			0.826	–
Yes	22 (25.88)	36 (28.35)		-0.056	13 (22.41)	14 (24.14)		0.039
No	63 (74.12)	91 (71.65)		0.056	45 (77.59)	44 (75.86)		-0.039

### 3.2 Weight change after preconception intervention

After the 16-week intervention, a statistically significant difference in weight change was observed between the groups (P < 0.001). The Target-oriented Group achieved a mean weight reduction of (9.96 ± 9.4)%, exceeding the predefined 8% target, whereas the Standard Lifestyle Group showed no significant change (0.68 ± 2.07) %; details are provided in **Table 2**. Further analysis of weight loss success revealed that the proportion of participants achieving ≥5% and ≥10% weight loss was significantly higher in the Target-oriented Group (60.34% and 43.10%, respectively) compared with the standard group (3.45% and 0; both P < 0.001). In the Target-oriented Group, the median weight reduction was 7.74% (IQR: 2.97–18.23%), reflecting a right-skewed distribution with a subset of high responders.

**Table 2:** Changes in body weight of patients in both groups at 16 weeks relative to baseline.

Indicator	Standard lifestyle group	Target-oriented group	t/χ^2^	*p*
Weight loss of patients at 16 week	0.68 ± 2.07	9.96 ± 9.40	-7.34	<0.001
Weight loss by 5% or more			43.217	<0.001
Yes	2 (3.45)	35 (60.34)		
No	56 (96.55)	23 (39.66)		
Weight loss by 10% or more			31.868	<0.001
Yes	0 (0)	25 (43.1)		
No	58 (100)	33 (56.9)		

### 3.3 Live birth and multiple pregnancy rates

The live birth rate was 39.66% (23/58) in the Target-oriented Group and 15.52% (9/58) in the standard group, with a risk difference of 24.1% (95% CI: 6.8 to 41.5%; P = 0.004); see **Table 3**. Among patients who achieved clinical pregnancy, the multiple pregnancy rate was 13.04% (3 of 23 clinical pregnancies) in the Target-oriented Group and 11.11% (1 of 9 clinical pregnancies) in the Standard Lifestyle Group (P > 0.05). The risk ratio of 2.55 (95% CI: 1.30–5.04), and a number needed to treat of 4.1, indicating that approximately 4 women would need to undergo the structured weight loss intervention for one additional live birth to be achieved.

**Table 3:** Comparison of live birth rate and multiple pregnancy rate between the two groups.

Indicator	Target-oriented group	Standard lifestyle group	Rate difference (95% CI)	χ^2^	*p*
Live birth			0.241(0.068, 0.415)	8.458	0.004
Yes	23 (39.66)	9 (15.52)			
No	35 (60.34)	49 (84.48)			
Multiple pregnancy			0.019(-0.286,0.247)	-	1.000
Yes	3 (13.04)	1 (11.11)			
No	20 (86.96)	8 (88.89)			

### 3.4 Time-to-pregnancy outcomes

**Figure 3:**
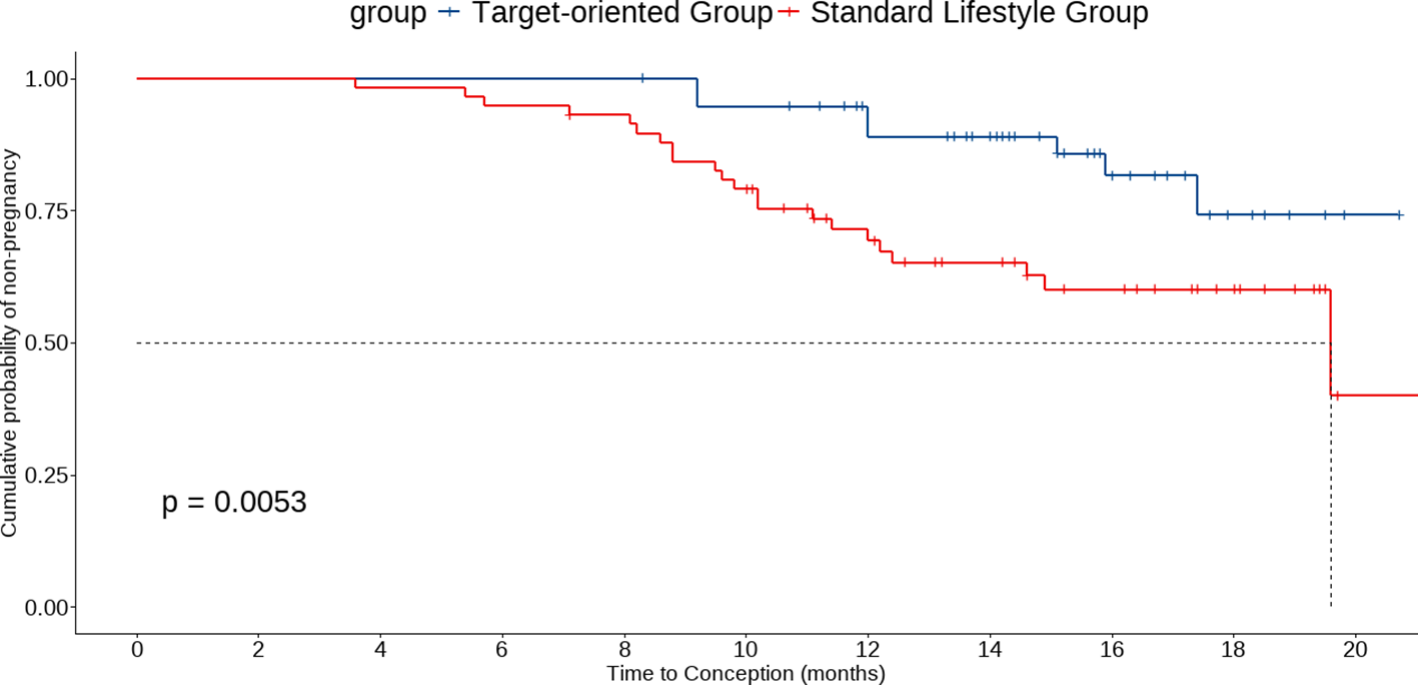
Kaplan-Meier curve for time to pregnancy in the two groups.

In the 16-week landmark cohort, Kaplan–Meier curves showed a higher cumulative incidence of clinical pregnancy in the Target-oriented Group compared with the Standard Lifestyle Group (log-rank P = 0.0053). In Cox proportional hazards regression from the landmark date, the Target-oriented Group had a higher hazard of achieving clinical pregnancy than the Standard Lifestyle Group, results were consistent after additional adjustment for baseline covariates (adjusted HR = 2.86, 95% CI: 1.31–6.22). Based on the Kaplan-Meier curves (**Figure 3**), the estimated median time to clinical pregnancy in the Target-oriented Group was 19.6 months (95% CI: 14.9 months — not reached), while the median was not reached in the Standard Lifestyle Group within the follow-up period, indicating that fewer than 50% of patients in the Standard Lifestyle Group achieved clinical pregnancy.

### 3.5 Neonatal outcomes and safety

Mean neonatal birth weight was (3.33 ± 0.36) kg in the Target-oriented Group and (3.40 ± 0.60) kg in the standard group (P > 0.05). The incidence of neonatal complications did not differ significantly between the groups (all P > 0.05); details are shown in **Table 4**.

**Table 4:** Comparison of fetal complications and neonatal birth weight between the two groups.

Indicator	Standard lifestyle group	Target-oriented group	*t/*χ^2^	*p*
Fetal complications			-	1.000
Yes	0 (0)	2 (6.45)		
No	10 (100)	29 (93.55)		
Fetal weight(kg)	3.4 ± 0.6	3.33 ± 0.36	0.458	0.65

## 4 Discussion

This retrospective cohort study was designed to evaluate whether a goal-directed weight loss intervention could improve live birth rates in women with obesity and infertility (BMI ≥30 kg/m²) planning to undergo ART, compared with proceeding directly to IVF without weight management. Based on the results, the Target-oriented Group achieved significantly greater weight loss than the Standard Lifestyle Group. The Target-oriented Group demonstrated a significantly higher live birth rate than the Standard Lifestyle Group. There were no significant differences in multiple pregnancy rates, neonatal birth weight, or neonatal complications.

We used time-to-event methods to evaluate differences in the timing of pregnancy achievement between strategies. Because the weight-loss intervention delayed ART initiation by design, we implemented a 16-week landmark analysis and analyzed time to clinical pregnancy from the landmark date to align follow-up windows and reduce immortal time bias.

These findings contrast with some previous studies. Several earlier reports indicated that although intensive weight loss interventions led to significant weight reduction in overweight women or women with obesity and infertility, they did not significantly improve pregnancy outcomes (11, 20). A 2016 Dutch RCT found no positive effect of weight loss intervention on the 2-year cumulative live birth rate among women with obesity and infertility (15). However, that study was limited by a modest between-group weight difference (3.3 kg) and a high discontinuation rate (22%) in the intervention group, which may have reduced the reliability of its conclusions (21). Our positive findings may be explained by several factors: (1) higher baseline BMI in our cohort (median 38.5 kg/m²) compared with previous studies, suggesting greater potential for metabolic improvement; (2) more intensive weight loss achieved (mean 9.96% vs. 3.3 kg in the Dutch RCT), exceeding the threshold needed for clinical benefit; (3) comprehensive intervention combining diet, exercise, and pharmacotherapy (orlistat), potentially more effective than lifestyle modification alone. It is important to acknowledge that the weight of randomized controlled trial evidence to date has not consistently supported a beneficial effect of preconception weight loss on ART outcomes. The Dutch RCT found no improvement in 2-year cumulative live birth rate (15). The Nordic RCT similarly failed to demonstrate a significant difference in live birth following the first IVF cycle (14). The FIT-PLESE RCT and the meta-analysis (11, 20) also reported null or modest effects. The positive findings of our study must therefore be interpreted in the context of this predominantly null RCT literature, and our results should be considered hypothesis-generating rather than confirmatory.

On the other hand, a growing body of evidence suggests that optimized weight loss strategies may positively influence ART outcomes. Palomba et al. (22) reported that regular physical activity in patients with obesity (BMI >30 kg/m²) significantly improved implantation rates (22.7%), clinical pregnancy rates (29.3%), and live birth rates (24.4%) compared with controls (6.9%, P < 0.001; 9.1%, P = 0.001; 7.4%, P = 0.004), irrespective of the amount of weight lost (23). In cases of severe obesity, bariatric surgery has been shown to improve live birth rates in subsequent ART cycles (24, 25). A systematic review by Sim et al. (26), which included 8 studies evaluating dietary, lifestyle, and surgical interventions, concluded that weight loss achieved through various methods may improve pregnancy and/or live birth rates in overweight or obese women. Excessive adiposity may lead to parasympathetic dysregulation, which can adversely influence hypothalamic–pituitary–ovarian axis function, ovarian responsiveness, and endometrial receptivity, ultimately impairing pregnancy achievement (10). The improved live birth rate following weight loss may be attributed to several mechanisms. Weight reduction improves insulin sensitivity, reducing hyperinsulinemia and compensatory hyperandrogenism, thereby restoring normal ovulatory function. Additionally, weight loss may enhance oocyte quality by reducing oxidative stress and mitochondrial dysfunction associated with obesity. The improved metabolic milieu may also optimize endometrial receptivity, facilitating embryo implantation (7). However, the optimal type and duration of weight loss intervention remain to be established through further prospective studies.

To our knowledge, few prior studies in this field have employed a Cox proportional hazards model with time-to-clinical-pregnancy as the primary time-to-event endpoint, combined with landmark analysis to address immortal time bias in a preconception weight loss cohort. Our time-to-event approach provides complementary evidence to the binary outcome analyses reported in most prior studies.

The substantial variability in weight loss observed in the Target-oriented Group (mean 9.96%, SD 9.40%; median 7.74%, IQR: 2.97–18.23%) warrants dedicated discussion, as it reflects the inherent heterogeneity of treatment response in a real-world clinical setting. This right-skewed distribution indicates the coexistence of high responders — a subset of patients achieving >15% weight reduction — and partial responders achieving <5% reduction, likely attributable to differences in baseline metabolic phenotype, dietary adherence, physical activity capacity, and psychological motivation. Importantly, rather than undermining the validity of our findings, this heterogeneity may in fact strengthen the clinical inference drawn from our study. The inclusion of patients with suboptimal adherence and modest weight loss in the Target-oriented Group would be expected to attenuate the observed between-group difference in live birth rates toward the null; the fact that a statistically and clinically significant benefit was nonetheless detected (risk difference 24.1%, 95% CI: 6.8–41.5%; P = 0.004) suggests that the true effect of optimal, goal-directed weight loss may be even greater than our overall estimates indicate. This interpretation is further supported by the observed trend toward a dose-response relationship, whereby patients achieving ≥10% weight reduction demonstrated numerically higher live birth rates compared with those achieving <10% reduction, although this comparison was underpowered and should be interpreted with caution. These findings highlight the need for future studies to identify baseline predictors of high versus low response to preconception weight management interventions, in order to enable precision-medicine approaches that optimize patient selection and maximize reproductive benefit.

Several limitations of this study deserve acknowledgment. First, the differential timing of IVF initiation between groups introduced the risk of immortal time bias, which we addressed by implementing a 16-week landmark analysis. However, this approach necessarily excludes patients who experienced very early clinical pregnancy or discontinued follow-up before week 16, potentially introducing directionally differential selection bias in each group and limiting generalizability to patients able and willing to defer ART initiation. Patients with more severe infertility diagnoses, diminished ovarian reserve, or advanced age may not be appropriate candidates for such a delay. Second, the retrospective, single-center design carries an inherently lower level of evidence than randomized controlled trials. Although the *post-hoc* power analysis indicated adequate power (>0.8) for the primary outcome, the sample size may be insufficient for subgroup analyses, and the single-center design may limit generalizability. Third, preference-based group allocation introduces the potential for confounding by indication. Women who voluntarily elected structured weight management may differ systematically from those who chose immediate IVF in unmeasured attributes such as health-related motivation, self-efficacy, adherence propensity, health literacy, and socioeconomic resources — none of which are captured by the propensity score model — and the observed benefit may partly reflect these selection effects rather than the causal effect of weight loss per se. Fourth, the absence of metabolic markers (fasting insulin, HOMA-IR, lipid profiles), ovarian reserve parameters, oocyte maturation rates, fertilization rates, embryo quality metrics, and endometrial thickness data precludes mechanistic interpretation of the observed differences in live birth rates and limits our ability to attribute the findings to specific biological pathways. Fifth, no formal assessment of psychological factors — including motivation, self-efficacy, depression, or anxiety — was performed, despite their plausible contribution to both weight loss magnitude and fertility outcomes given the preference-based allocation. Sixth, weight measurements relied on clinic-recorded values and may be subject to information bias; additionally, the diagnosis of binge eating disorder was based on the QEWP-R questionnaire without clinical interview confirmation, which may have led to patient misclassification. Seventh, neonatal outcome data were available for only 31 and 9 live births in the Target-oriented and Standard Lifestyle Groups, respectively; the study was not powered to detect differences in neonatal endpoints, and the descriptive statistics presented in **Table 4** should be regarded as exploratory only, with no conclusions regarding neonatal safety drawn from these data. Finally, the present study was limited by considerable participant attrition, with only 116 patients included in the final analysis from an initial cohort of 308. This high dropout rate was not was not comprehensively characterized and may introduce potential selection bias, thereby affecting the representativeness and generalizability of the results. Collectively, these limitations underscore the necessity of prospective randomized controlled trials with standardized allocation, comprehensive metabolic phenotyping, and formal psychological assessment to confirm the causal efficacy of preconception weight management in women with obesity and infertility planning to undergo ART.

## 5 Conclusion

This retrospective cohort study suggests that a 16-week intensive goal-directed weight loss intervention appeared to be associated with both significant weight reduction and higher live birth rates among women with obesity and infertility, in comparison with immediate IVF without prior weight management. While these findings are hypothesis-generating and clinically encouraging, the retrospective, single-center design and the possibility of residual confounding from unmeasured variables preclude definitive causal conclusions. Prospective randomized controlled trials incorporating standardized, goal-directed weight management protocols and achieving clinically meaningful weight reduction are needed to confirm and extend these findings.

## Data Availability

The raw data supporting the conclusions of this article will be made available by the authors, without undue reservation.
